# High Coverage and Low Utilization of the Double Fortified Salt Program in Uttar Pradesh, India: Implications for Program Implementation and Evaluation

**DOI:** 10.1093/cdn/nzaa133

**Published:** 2020-08-10

**Authors:** Shruthi Cyriac, Regine Haardörfer, Lynnette M Neufeld, Amy Webb Girard, Usha Ramakrishnan, Reynaldo Martorell, Mduduzi N N Mbuya

**Affiliations:** Doctoral Program in Nutrition and Health Sciences, Laney Graduate School, Emory University, Atlanta, GA, USA; Global Alliance for Improved Nutrition, Washington, DC, USA; Department of Behavioral Sciences and Health Education, Rollins School of Public Health, Emory University, Atlanta, GA, USA; Global Alliance for Improved Nutrition, Geneva, Switzerland; Doctoral Program in Nutrition and Health Sciences, Laney Graduate School, Emory University, Atlanta, GA, USA; Hubert Department of Global Health, Rollins School of Public Health, Emory University, Atlanta, GA, USA; Doctoral Program in Nutrition and Health Sciences, Laney Graduate School, Emory University, Atlanta, GA, USA; Hubert Department of Global Health, Rollins School of Public Health, Emory University, Atlanta, GA, USA; Doctoral Program in Nutrition and Health Sciences, Laney Graduate School, Emory University, Atlanta, GA, USA; Hubert Department of Global Health, Rollins School of Public Health, Emory University, Atlanta, GA, USA; Global Alliance for Improved Nutrition, Washington, DC, USA

**Keywords:** double fortified salt, implementation research, adaptive evaluation, coverage, adherence

## Abstract

**Background:**

Double fortified salt (DFS) is efficacious in addressing iron deficiency, but evidence of its effectiveness is limited. The few published evaluations do not include details on program implementation, limiting their utility for programmatic decisions.

**Objectives:**

We sought to characterize the coverage of a DFS program implemented through the Public Distribution System (PDS) in Uttar Pradesh, India, and understand the drivers of DFS adherence.

**Methods:**

After 8 mo of implementation, we surveyed 1202 households in 5 districts and collected data on sociodemographic characteristics, asset ownership, food security, and regular PDS utilization. We defined DFS program coverage as the proportion of PDS beneficiaries who had heard of and purchased DFS, and we defined DFS adherence as DFS use reported by households. We used principal component analysis to create an asset-based index of relative wealth, and we categorized households into higher/lower relative wealth quintiles. We conducted path analyses to examine the drivers of DFS adherence, particularly the mediated influence of household wealth on DFS adherence. The evaluation is registered with 3ie's Registry for International Development Impact Evaluations (RIDIE‐STUDY‐ID‐58f6eeb45c050).

**Results:**

The DFS program had good coverage: 83% of respondents had heard of DFS and 74% had purchased it at least once. However, only 23% exclusively used DFS. Respondents had low awareness about DFS benefits and considered DFS quality as poor. Being in a lower household wealth quintile and being food insecure were significant drivers of DFS adherence, and regular PDS utilization acted as a mediator. Adherence was lower in urban areas.

**Conclusions:**

We observed significant heterogeneity in DFS implementation as reflected by high coverage and low adherence. Findings from this process evaluation informed the design of an adaptive impact evaluation and provided generalizable insights for ensuring that the potential for impact is realized. Efforts are needed to increase awareness, improve product quality, as well as mitigate against the sensory challenges identified.

## Introduction

Addressing micronutrient deficiencies through food fortification is cost-effective (1), and one such successful strategy has been salt fortification to reduce iodine deficiency. Salt is commonly consumed, relatively affordable and accessible, and as such is an ideal fortification vehicle that can reach vulnerable populations. Leveraging this potential, researchers have considered since the 1960s using fortified salt to target iron deficiency and iron deficiency anemia (2), which have deleterious health, functional, and developmental implications. Indicated by lower iron stores in the body, iron deficiency often causes anemia (characterized by low hemoglobin concentrations). Although anemia has a complex multicausal etiology (3–7), iron deficiency is one of its common drivers in at least a fourth of the anemic population (8). Iron deficiency anemia is defined as meeting the criteria for both low iron stores (iron deficiency) and low hemoglobin (anemia) (9).

It is posited that countries with successful experience implementing universal salt iodization but struggling with high iron deficiency due to inadequate intakes may gain from adding iron to the salt. Double fortified salt (DFS) is the dual fortification of salt with iodine and iron that simultaneously targets both micronutrient deficiencies (10). The efficacy of DFS in reducing iron deficiency has been demonstrated in small controlled trials (11) that were conducted in India (12–15), Morocco (16), Côte d'Ivoire (17), and Ghana (18). However, the feasibility and effectiveness of DFS in a large-scale programmatic setting remain underexplored (19).

Only 2 studies have evaluated the effectiveness of DFS to date, highlighting a gap in evidence. Both studies were performed in Bihar, India. DFS distribution in 1 program was via school feeding programs (20), whereas the other program used social safety nets and retail markets as delivery platforms (21). Although these studies reported mixed results on DFS intervention impact, coverage levels were either unreported or low, and data on program implementation quality are limited. We conducted an evaluation of a DFS program in Uttar Pradesh (UP), India, to address the gaps in the current knowledge about program effectiveness.

### UP DFS program

India's National Family Health Survey 2015–2016 reported that in UP, 53% of nonpregnant women of reproductive age of 15–49 y (WRA) and 63% of preschool-aged children of 6–59 mo (PSC) were anemic (22). Several studies have also demonstrated a high prevalence of iron deficiency in this context (23). In 2016–2018, another survey specifically reported iron deficiency levels for children: in UP, 24% of young children (aged 1–4 y), 9% of school-aged children, and 17% of adolescents had low iron stores (24). To address this widespread anemia and iron deficiency, the UP government chose 10 districts with high anemia prevalence to distribute DFS using the Public Distribution System (PDS).

The PDS is a social safety net program in India, and PDS shops in UP distribute rations that include subsidized rice, wheat, and kerosene to eligible households every month. PDS eligibility is determined by the state government, and the lowest income households are categorized as Antyodaya Anna Yojana (AAY) cardholders, whereas slightly better off households are categorized as Priority Household (PHH) cardholders. After a recent restructuring of the PDS, 25% of rural households and 50% of urban households are not covered by the safety net program (25). DFS is subsidized for both AAY and PHH cardholders, and it is priced to be at least 3 times cheaper than iodized salt sold at an average price of INR 18/kg in the retail market. AAY cardholders receive DFS at INR 3/kg, and PHH cardholders receive DFS at INR 6/kg.

### Evaluation of the UP DFS program

The UP DFS program had an adaptive evaluation design that included baseline, midline (process evaluation), and endline assessments. Five of the 10 DFS program districts were chosen using simple random sampling for the evaluation. In conjunction with examining DFS effectiveness through baseline and endline assessments, the process evaluation specifically focused on assessing the implementation of this fortification strategy. We developed a program impact pathway and conducted a coverage survey. In this article, we present data on the coverage of the UP DFS program and assess the drivers of adherence. In addition, these data were used to determine whether to conduct the endline assessment for the DFS program, using an a priori evaluability threshold, decided based on similar fortification evaluations (26) in which at least 50% of the sampled population was utilizing DFS.

## Methods

### Sampling strategy

Twenty clusters of villages or wards (urban neighborhoods in India) were selected using stratified random sampling and population proportion to size for the baseline survey and revisited for the coverage survey, between November and December 2018, after 8 mo of DFS rollout. Of the 20 clusters, 5 each were randomly selected from villages and wards from within the entire district and 10 consisted of villages or wards randomly selected from border areas (within 20 km of the district border). Within each ward, 1 census enumeration block (CEB) was randomly selected using the 2011 Census of India data (27). After a mapping process, selected villages/CEBs were divided into 4 segments, and 3 households were selected from each segment to obtain 12 households from every cluster. Household selection from segments in villages took place by spinning a pen from a randomly selected landmark and assessing every fourth household in that direction for eligibility. Households in urban areas were selected using a similar segmentation approach, adapted from the multiple indicator cluster surveys (28).

The eligibility criteria for the baseline survey were maintained for the coverage survey. PDS cardholder households with at least 1 nonpregnant WRA and a PSC were interviewed; the primary respondent was the nonpregnant WRA. If more than 1 eligible WRA lived in the household, 1 was chosen randomly as the respondent. An additional household each was interviewed in 2 of the 5 districts due to oversampling, and these interviews were retained for the analyses, resulting in a final sample of 1202 interviews.

### Variable measurement

We assessed sociodemographic characteristics, housing conditions, asset ownership, and food security. We identified different salt types in household kitchens, distinguished based on packaging information: DFS from the PDS, iodized packaged salt from retail shops, or loose crystal salt from informal markets. We categorized caste—a symbol of social status—based on the classification provided by the Indian constitution, and we further categorized them into groups of higher and lower caste (29). Additional household-level assessments included PDS utilization, quality perceptions for PDS rations (defined using a 3-point Likert scale that rated “quality” as perceived by the respondent, categorizing it as “poor,” “good,” or “excellent”), and DFS stock holding. Interviews queried respondents’ levels of DFS awareness (**Supplemental Table 1**), and we considered responses that mentioned “good health or nutrition,” “anemia prevention,” or “goiter prevention,” as having at least partial awareness of DFS benefits.

We measured wealth using household assets as a proxy, and we used principal component analysis for variables representing 36 assets that included household goods and vehicles, livestock as well as property ownership, and attributes of housing structure such as construction quality and light and fuel sources to create household wealth quintiles. A dichotomous variable was created in which households in the lowest 2 quintiles constituted the lower wealth category, whereas the rest belonged to the higher wealth group.

Although PDS rations were available every month, some cardholder households did not regularly utilize the PDS for monthly purchase of subsidized rations. We therefore asked participants whether they regularly utilized their PDS cards and categorized households of those who responded affirmatively as regular PDS utilizers. We measured food insecurity using the Household Food Insecurity and Access Scale (30) and categorized households that were food secure and mildly food insecure as “food secure” and moderate/severe food insecure as “food insecure.” Participants of all households, including food-secure and mildly food-insecure households, worried about running out of food rarely, sometimes, or often, but only moderate and severely food-insecure households had to cut back on the quantity of food consumed.

**FIGURE 1:**
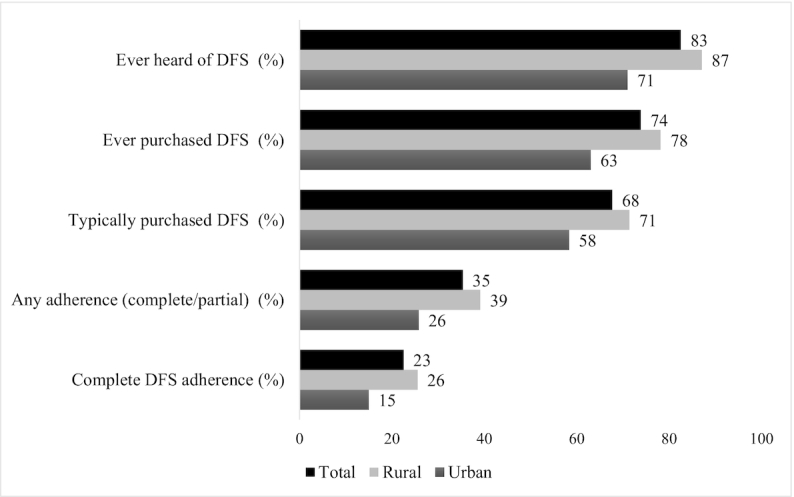
DFS program—coverage cascade, *n*  = 1202. Each indicator in this coverage cascade was conditional on having achieved the previous one. Households reporting that they typically purchased DFS bought DFS with their monthly purchase of rations in the Public Distribution System. Any DFS adherence included both households with “partial” and households with “complete” DFS adherence. Households with partial DFS adherence used DFS as a secondary salt, only in certain food or beverages. Households with complete DFS adherence used DFS in all foods and beverages that required salt. DFS, double fortified salt.

Adapting the Tanahashi framework on evaluating health service coverage, we examined a cascade of varying degrees of DFS program coverage (31–33). This coverage cascade indicated both DFS coverage and DFS adherence, and each indicator was conditional on having achieved the previous one (**Figure 1**). DFS coverage estimated the prevalence of those who had ever heard of DFS, had ever purchased DFS, and who purchased DFS with monthly PDS rations. To measure adherence, interviewed households listed all salt types in kitchens and their usage in food and beverages. “Any DFS adherence” included 2 subsets of households: households with “partial DFS adherence” and households with “complete DFS adherence.” Partial DFS adherence was used to categorize households reporting DFS as a secondary salt, used only in certain dishes or drinks, whereas complete DFS adherence was used for households that reported exclusive DFS use in foods and beverages.

### Hypothesized pathways

**FIGURE 2:**
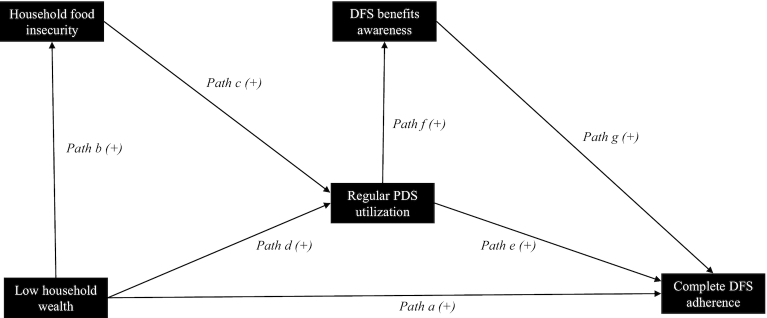
Hypothesized path model indicating measured variables, pathways, and the direction of effect. Measured predictors are shown in boxes; hypothesized pathways are labeled and shown by the arrows. All hypothesized pathways have a positive direction of effect. Wealth quintiles were created, and low household wealth included the lower 2 wealth quintiles; wealth was measured using household assets as proxy. The Household Food Insecurity and Access Scale (30) was used to identify moderately/severely food-insecure households, which were categorized as experiencing household food insecurity. Households purchasing monthly PDS rations were noted to have regular PDS utilization. Households that had at least a partial understanding about DFS being beneficial for “good health or nutrition”/“anemia prevention”/“goiter prevention” were categorized as having DFS benefits awareness. Households with complete DFS adherence used DFS in all foods and beverages that required salt. DFS, double fortified salt; PDS, Public Distribution System.

For all path analyses, the outcome variable of interest was complete DFS adherence because it was most likely to capture the DFS program's potential to benefit. We examined the relation between household wealth and complete DFS adherence and theorized a path model (**Figure 2**), which proposed that regular PDS utilization, household food insecurity, and DFS awareness mediated this relation.

Our fieldwork experience indicated that despite being cardholders, several households could not access the PDS due to an ongoing restructuring of the safety net program, including installation of a biometric system and linking PDS cards to a national identity card (25). We also noted that PDS rations were sold as a bundle, and many had to purchase DFS in order to get grains and kerosene fuel. Some households that purchased DFS as part of these bundled sales used it for own consumption, whereas others simply stored it, mixed it in cattle feed, or donated it to neighbors who used DFS. It was likely that some lower wealth cardholder households faced constraints to regular PDS utilization and accepted DFS donations, it being cheaper than purchasing alternate salt. Therefore, we hypothesized that lower wealth nonregular PDS utilizers are more likely to use DFS compared with higher wealth nonregular PDS utilizers, indicating a link between lower wealth in households and complete DFS adherence—that is, exclusive DFS use in foods (Figure 2, path a).

In addition, we hypothesized that household food insecurity and regular PDS utilization would mediate the relation between household wealth and complete DFS adherence. First, we expect lower wealth households to experience food insecurity (Figure 2, path b) and that greater food insecurity may lead to regular PDS utilization (Figure 2, path c). Second, we expect lower wealth households to rely on the PDS for kerosene fuel, in addition to the food grains (Figure 2, path d). Not all those who purchased DFS completely adhered to it due to quality concerns. However, we hypothesize regular PDS utilizer households to be more likely to use DFS as they accumulate it from monthly purchases (Figure 2, path e). Finally, we expect that regular PDS utilizers will be exposed to DFS messaging by shop owners (Figure 2, path f), and households aware of DFS benefits may be more adherent (Figure 2, path g). DFS program staff provided a 1-time training to PDS shop owners, describing DFS contents and demonstrating their benefits for preventing anemia and goiter. PDS shop owners were asked to verbally communicate about DFS and its benefits to household members who came to purchase rations.

### Analyses

We used descriptive analyses to assess individual characteristics, such as respondent age and education levels, and household characteristics, including family size, primary income source, access to facilities, religion, and caste. We examined and retained all outlier values after scrutinizing related variables and determining plausibility. Examination of bivariate associations and confounding assessment helped build the final path model, adjusted for household religion and household head's education. We examined all variables in the path model for missing data and found that values for household head's education were missing for 11 interviews. We adopted a listwise deletion approach to account for missing data (34) after determining the values to be missing at random. We expected pathways to vary based on location of residence, and we created separate household wealth quintiles for rural and urban areas to examine location-specific path models (**Supplemental Table 2**, panel A). Except for 1 ordinal variable (household head education), all other variables in the path model were dichotomous.

We performed preliminary analyses using SAS software version 9.4 and weighted path analyses in R version 3.5.3 (R Foundation for Statistical Computing). We utilized survey methods and structural equation modeling approaches (R lavaan and lavaan.survey packages, versions 0.6–5 and 1.1.3.1), adopting the diagonally weighted least-squares method to account for both ordered and dichotomous variables to conduct a path analysis. All standardized coefficients were exponentiated to obtain the adjusted OR (aOR); indirect and total effects were calculated. We assessed goodness of model fit using chi-square, root mean square error of approximation (RMSEA), comparative fit index (CFI), and standardized root mean squared residual (SRMR). The RMSEA, CFI, and SRMR suggest reasonably good model fit when they are <0.08, >0.90, and <0.08, respectively (35, 36).

### Ethical considerations

Institutional review boards at Sanjay Gandhi Post Graduate Institute of Medical Sciences and Emory University reviewed and approved the data collection and analyses protocol. The evaluation is registered with 3ie's Registry for International Development Impact Evaluations (RIDIE-STUDY-ID-58f6eeb45c050).

## Results

Sociodemographic characteristics of the sample are presented in **Table 1**, stratified by location of residence. The majority of respondents were Hindu, resided in rural areas, and belonged to a lower caste. Most respondents lived with their extended family, and household size averaged 7 members. Overall education level was low, but the proportion of respondents with a college degree was ∼1.5 times higher in urban compared with rural areas (*P* < 0.001). Urban households were also more likely to have access to electricity, piped water, and gas stoves. Nearly half the rural households experienced moderate to severe food insecurity (**Supplemental Table 3**). We also noted that more households in rural areas regularly utilized the PDS compared with those in urban settings. Two-thirds of sampled households found DFS quality to be poor, but a higher proportion of urban households noted the quality of PDS rice and DFS to be poor.

**TABLE 1:** Descriptive analysis of household and individual characteristics, stratified by location of residence^1^

	Total		Rural		Urban		
Characteristic	%	*n* (1202)	%	*n* (861)	%	*n* (341)	*P* value^2^
Average age of respondent	27.9 ± 4.6	1202	27.5 ± 4.6	861	28.8 ± 4.5	341	<0.001
Religion—Hindu, %	84.53	1016	90.48	779	69.5	237	<0.001
Caste—higher, %	76.27	916	73.49	632	83.28	284	<0.001
Respondent's education, %							<0.001
No education	28.55	342	29.05	249	27.27	93	
Primary or middle school	28.13	337	29.4	252	24.93	85	
Secondary or high school	32.2	386	33.96	291	27.86	95	
Graduate level or higher	11.1	133	7.58	65	19.94	68	
Household head's education, %							<0.001
No education	26.6	317	30.18	258	17.56	59	
Primary or middle school	24.4	291	24.91	213	23.21	78	
Secondary or high school	28.6	340	27.37	234	31.55	106	
Graduate level or higher	20.4	243	17.54	150	27.68	93	
Average household size	7.1 ± 3.0	1202	7.1 ± 3.0	861	6.9 ± 2.9	341	0.221
Households living with extended family, %	60.7	730	61.44	529	58.94	201	0.424
Main income source—nonagriculture, %	67.2	808	57.26	493	92.38	315	<0.001
Primary light source—electricity, %	82.6	962	78.37	652	93.09	310	<0.001
Primary fuel source—LPG/natural gas, %	39.4	473	22.65	195	81.52	278	<0.001
Primary water source, %							<0.001
Tube well/bore well	78.4	942	87.22	751	56.01	191	
Piped into dwelling or yard	9.2	149	5.9	51	28.7	98	
Regularly utilizes PDS, %	80.2	964	84.2	725	70.09	239	<0.001
Perception of PDS ration quality, %							
Poor rice quality	17.36	155	14.52	99	26.54	56	<0.001
Poor wheat quality	6.71	61	6.46	44	7.46	17	0.732
Poor kerosene quality	1.84	12	1.69	9	2.52	3	0.587
Poor DFS quality	64.66	525	61.66	378	73.87	147	0.005
Households with any DFS stock currently present, %	55.66	669	60.74	523	42.82	146	<0.001
Average no. of salt types present in household	1.4 ± 0.5	1202	1.4 ± 0.5	861	1.3 ± 0.5	341	0.001

1 Values are means ± SDs or percentages (%). DFS, double fortified salt; LPG, liquefied petroleum gas; PDS, Public Distribution System.

2 *P* value indicates difference between rural and urban estimates.

Figure 1 shows the DFS program coverage cascade. Rural areas had higher DFS coverage and adherence compared with urban settings. Among overall survey participants, 83% either had ever seen DFS packets or had ever heard of the product; 74% of all participants had purchased DFS at least once, and 68% reported purchasing it every month from the PDS along with the other PDS commodities. Regarding DFS adherence, 35% of the survey sample reported any adherence (i.e., using DFS either partially or completely), and 23% reported complete adherence. There were district level variations in DFS adherence (**Supplemental Table 4**), with 2 districts indicating higher adherence especially in the rural areas.

**FIGURE 3:**
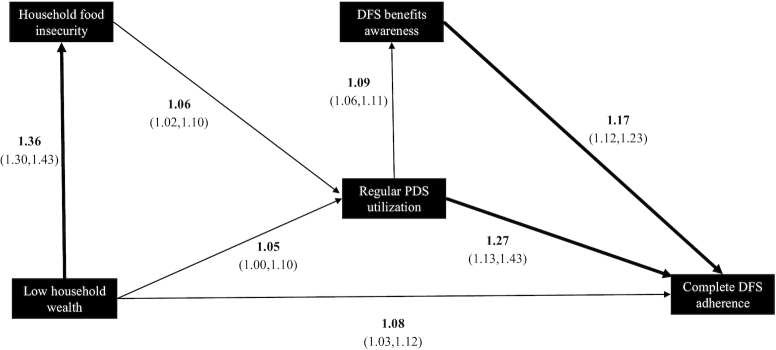
Rural path model showing standardized direct effects (*n* = 861). Only standardized direct effects between the measured predictors and dependent outcomes are shown. All direct effects have been exponentiated to obtain the adjusted ORs, and 95% CIs are indicated in parentheses. Standardized indirect effects indicating mediated paths are not shown, but they are presented in Table 2. Thicker lines denote an OR >10%; Model fit indices showed a good fit for this path model: *P* value (chi-square) = 0.003, RMSEA = 0.07, CFI = 0.99, and SRMR = 0.01. CFI, comparative fit index; DFS, double fortified salt; PDS, Public Distribution System; RMSEA, root mean square error of approximation; SRMR, standardized root mean squared residual.

After removing the 11 households with missing information on household head education, the final sample for path models was 1191 households. Findings from the path model are illustrated in **Figure 3**. The model fit for rural sample were all within acceptable range [*P* value (chi-square) = 0.003, RMSEA = 0.07, CFI = 0.99, and SRMR = 0.01] (35, 36), and no model respecification was required. However, the urban model did not show a good fit [*P* value (chi-square) < 0.001, RMSEA = 0.35, CFI = 0.99, and SRMR = 0.02]. Subsequent model respecifications of the urban model based on theoretically vetted modification indices resulted in nonconvergence, and we therefore do not present the urban model in this article.

**Table 2** describes direct, indirect, and total effects separately for total and rural models. With the overall sample size of path models containing a majority of rural households (*n* = 861), the effects in the total sample were qualitatively similar to the effects in rural models. We discuss only the rural effects here; the direct effects for the total model are presented in **Supplemental Figure 1**. Standardized direct effects in the path model for the rural sample (Figure 3) indicated that households in lower wealth quintiles had greater odds of complete DFS adherence (aOR: 1.08; 95% CI: 1.03, 1.12); they also had greater odds of experiencing household food insecurity (aOR: 1.36; 95% CI: 1.30, 1.43). Odds of regular PDS utilization increased with greater household food insecurity (aOR: 1.06; 95% CI: 1.02, 1.10) and with lower household wealth (aOR: 1.05; 95% CI: 1.00, 1.10). Regular PDS utilization increased the odds of improved DFS awareness (aOR: 1.09; 95% CI: 1.06, 1.11). Improved awareness and regular PDS utilization had a positive direct effect on DFS use. Significant indirect paths from household wealth to DFS adherence in the rural model (Table 2) indicated mediation by regular PDS utilization, through household food insecurity, showing increased overall odds (Table 2) of DFS adherence (aOR: 1.28; 95% CI: 1.13, 1.44). The mediated path from PDS utilization and DFS awareness was not significant, and the total effect remained the same as the direct effect seen in Figure 3 (aOR: 1.17; 95% CI: 1.12, 1.23).

**TABLE 2:** Drivers of complete DFS adherence: adjusted path model showing standardized coefficients of direct and indirect pathways through which lower wealth households adhere to DFS use in all foods^1^

		Total model (*n* = 1191)				Rural model (*n* = 861)			
Paths and dependent outcomes	Measured predictor	Direct effect	Indirect effect	Total effect	aOR (95% CI)	Direct effect	Indirect effect	Total effect	aOR (95% CI)
Complete DFS adherence (path a)	Low HH wealth	0.068		0.07	1.07 (1.02,1.12)	0.074		0.07	1.08 (1.03,1.12)
HH FIS (path b)	Low HH wealth	0.299		0.3	1.35 (1.28,1.42)	0.308		0.31	1.36 (1.30,1.43)
Regular PDS utilization (path c)	HH FIS	0.071		0.07	1.07 (1.03,1.12)	0.057		0.06	1.06 (1.02,1.10)
Regular PDS utilization (path d)	Low HH wealth	0.048		0.05	1.05 (1.00,1.10)	0.048		0.05	1.05 (1.00,1.10)
DFS benefits awareness (path f)	Regular PDS utilization	0.062		0.06	1.06 (1.03,1.10)	0.083		0.08	1.09 (1.06,1.11)
Complete DFS adherence (path e)	Regular PDS utilization	0.244				0.239			
Through regular PDS utilization(paths d and e)	Low HH wealth		0.012				0.011		
Through HH FIS and regular PDSutilization (paths b, c, and e)	Low HH wealth		0.005	0.25	1.28 (1.10,1.49)		0.004	0.24	1.28 (1.13,1.44)
Complete DFS adherence (path g)	DFS benefits awareness	0.148				0.157		0.16	
Through DFS benefits awarenessand regular PDS utilization(paths d, f, and g)	Low HH wealth		0.000				0.001		
Through DFS benefits awareness,regular PDS utilization, and HHFIS (paths b, c, f, and g)	Low HH wealth		0.000	0.15	1.16 (1.11,1.21)		0.000	0.16	1.17 (1.12,1.23)

1 aOR, adjusted OR; DFS, double fortified salt; FIS, food insecurity; HH, household; PDS, Public Distribution System.

## Discussion

After 8 mo of implementation, the UP DFS program had attained high coverage but low adherence. Although bundling DFS sales with subsidized grains ensured continued DFS purchase, product quality concerns resulted in low DFS adherence. In another DFS program in Bihar, DFS use decreased over time, with many users trying and giving up DFS after finding black specks in food. Subsequently, null findings were reported when the evaluators conducted an impact assessment in Bihar (21). In UP, we conveyed the remedial measures to program staff in real time, focusing on actions to improve DFS adherence through better product quality and increased awareness about DFS benefits.

Rural households were likely to be more impoverished than urban households. A combined wealth index created for the overall population (Supplemental Table 2, panel B) showed urban households to be largely concentrated in the higher 2 relative wealth quintiles. Compared with rural households, urban households relied less on subsidized rations and were more likely to self-select themselves out of the PDS. Our rural path model indicates that lower household wealth, regular PDS utilization, and DFS awareness were strong drivers of DFS use. Building awareness around DFS benefits worked in this context, in which DFS adherence levels were higher among those who positively perceived DFS.

These findings reveal 3 lessons for the UP DFS program. First, there is a need to overcome DFS product limitations and address quality concerns raised by users. DFS production is complex, with formulations for iron compounds constantly evolving. Four iron formulations for DFS currently exist (10), and the UP program tested 1 of these, which showed promise of addressing the discoloration problems faced in other DFS trials. However, as the program rolled out, DFS quality was compromised due to the significant investments required to produce and blend the iron formulation with high-grade refined salt, high production costs, and a lack of standards for producing extruded iron compounds (37). In our qualitative assessments (results forthcoming), DFS users raised concerns about sensory changes in food, and nonadherent households likely valued product quality over price subsidies or perceived benefits. Similar sensory changes in food were noticed by participants in a consumer acceptability study conducted by the UP DFS program in New Delhi, India (38), testing the same formulation. The study reported that DFS caused varying levels of discoloration in food, which depended on heating methods used during preparation (boiling, pressure cooking, and sautéing), with almost no change noticed in food that was prepared with no heat (e.g., salads and cold beverages). However, cooking methods, the types of dishes prepared, and the timing of meals can vary regionally and ultimately influence DFS adherence, highlighting the need to invest in context-specific sensory trials.

Second, the DFS program should reinforce efforts to boost awareness regarding the safety and benefits of DFS. Awareness creation efforts in the New Delhi DFS sensory trial showed that >85% of the participants were willing to use DFS after learning about its benefits (38). Although improving product quality is paramount, an interim strategy could be to proactively inform users to anticipate sensory changes in food due to DFS, and create awareness about the reasons for these changes, such that they consider discoloration in food as a signal of nutritional value. Similar programs that distributed micronutrient powders in multiple contexts have successfully adopted such a communication strategy, in which users readily accepted the intervention once they were aware of what to expect and convinced about product safety and benefit (39–41).

Finally, it is important to consider strategies to expand DFS access and availability in urban areas, where 50% of the population is not covered by the PDS. Simultaneously, the need for complementary strategies, including non-DFS interventions (42, 43), should be recognized to address state-level issues of iron deficiency and iron deficiency anemia. Although it is reassuring to note that food-insecure, lower wealth households are getting access to DFS, iron deficiency and iron deficiency anemia are not restricted only to the poorer rural households in India (42, 44, 45). Concurrent efforts such as expanding the DFS distribution through retail markets or liaising with other iron-fortification initiatives with a larger reach (46, 47)—for example, wheat fortification—might ensure that all populations suffering from iron deficiency or dietary inadequacy are reached.

These findings must be interpreted within the limitations of this study. There could be a social desirability bias—in either direction—for questions regarding PDS purchases and product quality rating because we informed respondents prior to interviews that we were evaluating the DFS program to improve it. Second, due to the cross-sectional study design, it is difficult to establish temporal relations with household wealth and food security. Third, the urban model fit was poor, indicating the possibility of an unmeasured mechanism and pointing to the need for more research, including qualitative interviews, to understand other potential drivers of DFS adherence in urban contexts—for example, increased access to retail markets. Fourth, although bundling of DFS with PDS rations was reported by the sampled population, the coverage survey questionnaire did not adequately capture this information to quantify the exact prevalence of bundling. However, we were able to use this insight to further examine PDS bundling in our qualitative assessments. Despite these limitations, the path models and coverage analyses provide important lessons for program implementers to improve coverage and adherence of DFS. Although not generalizable to all DFS programs and contexts, this evaluation of the UP DFS program has broader implications for the design and implementation of any nutrient intervention, especially those that use social safety nets as delivery platforms.

Insights on implementation fidelity are critical to interpret findings and inform the design of future evaluations. We used the results to inform programmatic course correction and assess the evaluability of the program (26). Examining the DFS program in a real-world context and identifying inefficiencies in program delivery helped assess the adequacy of the program. It elucidated the extent to which the program is moving in the expected direction, and it provided the opportunity for course correction, before moving on to conduct the endline evaluation. The coverage survey indicated districts that met the endline evaluability threshold (Supplemental Table 4). It informed the selection of districts for the endline evaluation (48, 49), modifying it to focus on rural areas in the 2 districts that had more adherents.

Our process and findings provide important information for conducting and using implementation research for designing and evaluating nutrient interventions at scale. At this stage of the UP program, the potential for measurable benefits is constrained by the low rates of DFS adherence. This study reveals implementation issues that the UP program and DFS programs globally must address. Targeted efforts to improve adherence are needed: addressing product quality issues, investing in well-designed awareness campaigns that ensure behavior change (50), and strengthening DFS program delivery can help DFS programs achieve the desired impacts on reducing iron deficiency and iron deficiency anemia.

## Supplementary Material

nzaa133_Supplemental_File

## Notes

Funding for this review was provided by the Bill & Melinda Gates Foundation.

Author disclosures: The authors report no conflicts of interest.

Supplemental Table 1–4 and Supplemental Figure 1 are available from the “Supplementary data” link in the online posting of the article and from the same link in the online table of contents at http://academic.oup.com/cdn/.

Abbreviations used: AAY, Antyodaya Anna Yojana; aOR, adjusted OR; CEB, census enumeration block; CFI, comparative fit index; DFS, double fortified salt; PDS, Public Distribution System; PHH, Priority Households; PSC, preschool-aged children; RMSEA, root mean square error of approximation; SRMR, standardized root mean squared residual; UP, Uttar Pradesh; WRA, women of reproductive age.
