# Supplementary material for: High Coverage and Low Utilization of the Double Fortified Salt Program in Uttar Pradesh, India: Implications for Program Implementation and Evaluation
Source: Curr Dev Nutr. 2020 Aug 10;4(9):nzaa133. doi: 10.1093/cdn/nzaa133 (PMC7455457; doi:10.1093/cdn/nzaa133)
Supplement: nzaa133_Supplemental_File [file nzaa133_supplemental_file.docx]

**Supplementary Table 1: Awareness levels about DFS contents**

|  | **Rural** | | **Urban** | |
| --- | --- | --- | --- | --- |
|  | Frequency (%) | N (861) | Frequency (%) | N (341) |
| Good for health and nutrition | 9.5 | 82 | 13.8 | 47 |
| Mentions both Anemia/related issues and Goiter/related issues | 2.3 | 20 | 5.9 | 20 |
| Only mentions Goiter/iodine related issues | 1.5 | 13 | 2.9 | 10 |
| Only mentions Anemia/iron related issues | 0.9 | 8 | 0.6 | 2 |
| Not Aware | 85.7 | 738 | 76.8 | 262 |

**Supplementary Table 2: Wealth quintile creation for separate (panel A) and combined (panel B) sample populations**

| Panel A: Separate wealth quintiles created for urban (N=341) and rural (N-861) sample population | | | | | |
| --- | --- | --- | --- | --- | --- |
|  | Urban | | Rural | |  |
| Wealth Quintiles | % | N | % | N | Total N |
| Lowest | 19.9 | 68 | 20.0 | 172 | 240 |
| Low | 19.9 | 68 | 20.0 | 172 | 240 |
| Middle | 20.2 | 69 | 20.1 | 173 | 242 |
| High | 19.9 | 68 | 20.0 | 172 | 240 |
| Highest | 19.9 | 68 | 20.0 | 172 | 240 |
| Total | 100 | 341 | 100 | 861 | 1202 |
| Panel B: Composite wealth quintile created for total sample population (N=1202) | | | | | |
|  | Urban | | Rural | |  |
| Wealth Quintiles | % | N | % | N | Total N |
| Lowest | 2.6 | 9 | 26.8 | 231 | 240 |
| Low | 5.0 | 17 | 26.0 | 224 | 241 |
| Middle | 14.1 | 48 | 22.3 | 192 | 240 |
| High | 29.3 | 100 | 16.4 | 141 | 241 |
| Highest | 49.0 | 167 | 8.5 | 73 | 240 |
| Total | 100 | 341 | 100 | 861 | 1202 |

**Supplementary Table 3: Food Security by rural and urban areas**

|  | **Rural** | | **Urban** | |
| --- | --- | --- | --- | --- |
|  | Frequency (%) | N (861) | Frequency (%) | N (341) |
| Food secure | 37.63 | 324 | 51.03 | 174 |
| Mild food insecurity | 13.01 | 112 | 12.32 | 42 |
| Moderate food insecurity | 37.4 | 322 | 28.74 | 98 |
| Severe food insecurity | 11.96 | 103 | 7.92 | 27 |
| Total | 100 | 861 | 100 | 341 |

**Supplementary Table 4: District-level DFS adherence rates (complete or any adherence) in rural areas (N=861)**

|  | DFS complete adherence | | DFS any adherence | | N = 861 |
| --- | --- | --- | --- | --- | --- |
| District Name | Yes (%) | No (%) | Yes (%) | No (%) |  |
| Moradabad | 19.1 | 81.0 | 25.6 | 74.4 | 168 |
| Etawah | 37.4 | 62.6 | 54.6 | 45.4 | 163 |
| Auraiya | 41.1 | 58.9 | 56.1 | 43.9 | 180 |
| Faizabad | 17.7 | 82.3 | 29.3 | 70.7 | 181 |
| Mau | 12.4 | 87.6 | 30.2 | 69.8 | 169 |

*Note: Complete adherence is DFS use in all foods, any adherence is partial DFS use in some foods or complete DFS use in all foods. An a priori threshold of 50% adherence was selected for selection of a district for endline evaluation. No district met the cut-off for ‘complete DFS adherence’, two districts - Etawah and Auraiya - met 50% cut-off for ‘any DFS adherence’*

**Supplementary Figure 1: Path Model for the overall sample (N=1191)**


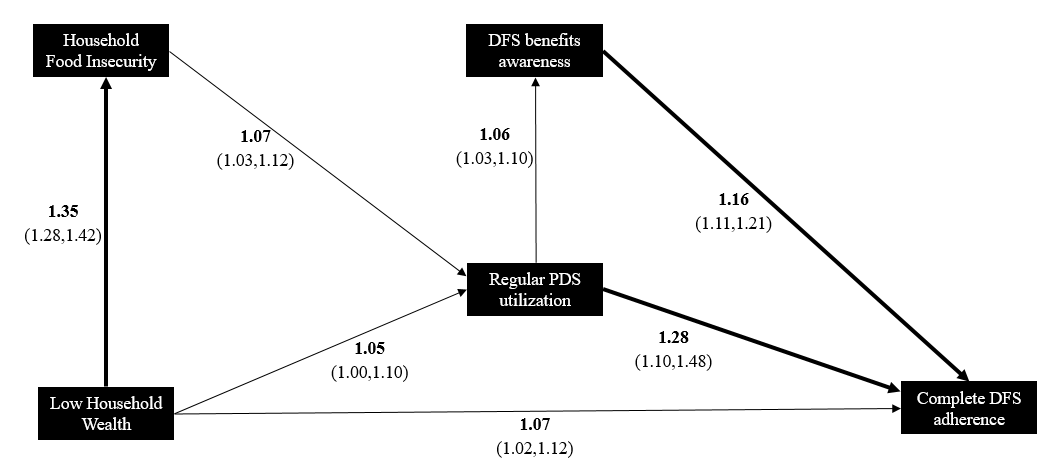


Standardized direct effects are shown in the figure; standardized indirect effects are listed in Table 2. Thicker lines denote an odds greater than 10%; Model Fit: P-value (Chi-square):0.004, RMSEA: 0.05, CFI: 0.99, SRMR: 0.01
